# Design and Engineering of an Efficient Peroxidase Using Myoglobin for Dye Decolorization and Lignin Bioconversion

**DOI:** 10.3390/ijms23010413

**Published:** 2021-12-30

**Authors:** Wen-Jie Guo, Jia-Kun Xu, Sheng-Tao Wu, Shu-Qin Gao, Ge-Bo Wen, Xiangshi Tan, Ying-Wu Lin

**Affiliations:** 1 School of Chemistry and Chemical Engineering, University of South China, Hengyang 421001, China; 20192005210167@usc.edu.cn (W.-J.G.); wushengtao@usc.edu.cn (S.-T.W.); 2 Key Laboratory of Sustainable Development of Polar Fisheries, Ministry of Agriculture and Rural Affairs, Yellow Sea Fisheries Research Institute, Chinese Academy of Fishery Sciences, Laboratory for Marine Drugs and Byproducts of Pilot National Laboratory for Marine Science and Technology, Qingdao 266071, China; xujk@ysfri.ac.cn; 3 Key Laboratory of Protein Structure and Function of Universities in Hunan Province, University of South China, Hengyang 421001, China; 2014001853@usc.edu.cn (S.-Q.G.); wengebo@usc.edu.cn (G.-B.W.); 4 Department of Chemistry & Institute of Biomedical Science, Fudan University, Shanghai 200433, China; xstan@fudan.edu.cn

**Keywords:** heme enzymes, protein design, dye-decolorizing peroxidase, kraft lignin bioconversion

## Abstract

The treatment of environmental pollutants such as synthetic dyes and lignin has received much attention, especially for biotechnological treatments using both native and artificial metalloenzymes. In this study, we designed and engineered an efficient peroxidase using the O_2_ carrier myoglobin (Mb) as a protein scaffold by four mutations (F43Y/T67R/P88W/F138W), which combines the key structural features of natural peroxidases such as the presence of a conserved His-Arg pair and Tyr/Trp residues close to the heme active center. Kinetic studies revealed that the quadruple mutant exhibits considerably enhanced peroxidase activity, with the catalytic efficiency (*k*_cat_/*K*_m_) comparable to that of the most efficient natural enzyme, horseradish peroxidase (HRP). Moreover, the designed enzyme can effectively decolorize a variety of synthetic organic dyes and catalyze the bioconversion of lignin, such as Kraft lignin and a model compound, guaiacylglycerol-β-guaiacyl ether (GGE). As analyzed by HPLC and ESI-MS, we identified several bioconversion products of GGE, as produced via bond cleavage followed by dimerization or trimerization, which illustrates the mechanism for lignin bioconversion. This study indicates that the designed enzyme could be exploited for the decolorization of textile wastewater contaminated with various dyes, as well as for the bioconversion of lignin to produce more value-added products.

## 1. Introduction

In the last decade, the environmental pollution caused by organic chemicals has received much attention. The main sources of organic matter in wastewater are synthetic dyes from the textile industry [1] and lignin from the paper industry [2]. For example, various dyes such as anthraquinones, azo, and triphenylmethane compounds in wastewater threaten aquatic organisms and human health [3]. Meanwhile, lignocellulosic biomass has great potential as raw material to produce more value-added products such as glucose, fuel, and furfural through biologically refining [4]. Therefore, it is important to effectively degrade organic dyes and utilize lignin in wastewater for environmental treatment.

At present, chemical methods are most used to purify or degrade synthetic dyes and lignin in polluted water. The main degradation methods include physical processes such as membrane filtration, precipitation, flotation, adsorption, and ion exchange, and chemical processes such as electrolysis and chemical reduction/oxidation [5]. However, due to its complex structure, amorphous and non-uniform nature, the degradation of lignin generally occurs at a high temperature (200–500 °C) [6]. On the other hand, the addition of catalysts may reduce the temperature required for the reaction. For example, the conversion of lignin may occur at 200 °C via a fragmentation−hydrogenolysis process with Ni/C catalyst [7].

In addition to chemical and physical methods, biotechnological treatments use both native and artificial metalloenzymes such as heme enzymes and others [8,9,10,11,12,13]. Comparatively, the biologically catalytic process often occurs under mild reaction conditions. Both organic dyes and lignin in wastewater could be degraded by bacteria and fungi. The main functional enzymes include dye-decolorizing peroxidase (DyPs), lignin peroxidase, manganese peroxidase, and versatile peroxidases in these organisms [14,15,16]. Recently, the use of enzymes in organisms to catalyze degradation is the focus of the research. As found in eukaryotes and prokaryotes, the heme-containing peroxidases use H_2_O_2_ as an electron acceptor to catalyze the oxidation of a large number of substrates [17,18]. Moreover, other metalloenzymes such as laccase and *β*-esterase can also play a catalytic role in biodegradation [19].

Notably, recent studies have shown that DyPs show great potential for degrading lignin and a variety of synthetic dyes, making them potentially useful in wastewater bioremediation [20]. DyPs are classified into four subfamilies (A-D) according to their sequence characteristics. Generally, different types of DyPs have different catalytic properties toward lignin model compounds [21,22]. There are also reports suggesting the mechanism of how A-type DyPs oxidize bulky dye molecules such as reactive blue 19 (RB19) and lignin polymers [23]. These achievements highlight the potential of DyPs as biocatalysts while they work at relatively low pH values (pH 3–4) [24].

**Figure 1:**
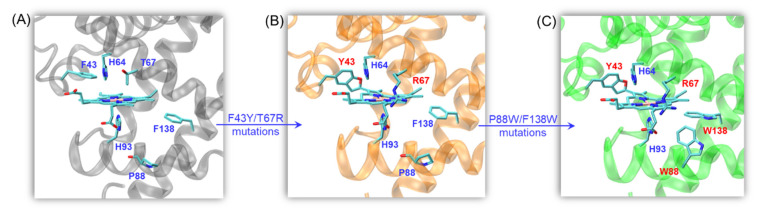
(**A**) X-ray crystal structure of wild-type (WT) Mb (PDB code 1JP6[35]) showing the heme active site; (**B**) X-ray crystal structure of F43Y/T67R Mb double mutant showing the double Tyr-heme cross-links (PDB code 6JP1[26]); (**C**) The modeling structure of F43Y/T67R/P88W/F138W Mb quadruple mutant showing the location of Trp88 and Trp138.

To design efficient heme peroxidases, we used the O_2_ carrier myoglobin (Mb) as a model protein by modification of the heme center (Figure 1A). For example, by introducing a distal Tyr residue (F43Y mutation), we discovered a new post-translational modification in F43Y Mb that forms a novel Tyr-heme cross-link [25]. To mimic the heme active site of natural peroxidases containing a conserved His-Arg pair more closely, we constructed a double mutant F43Y/T67R Mb by further introducing a distal Arg67 in F43Y Mb (Figure 1B), which improves the protein stability and significantly enhances the peroxidase activity [26]. Recently, we applied the double mutant F43Y/T67R Mb for the bioconversion of lignin with satisfying results [27]. Moreover, natural DyPs contain multiple Tyr and Trp residues responsible for electron transfer in dye decolorization [28,29]. Inspired by this structural feature, in previous studies, we constructed two triple mutants F43Y/T67R/F138W Mb (termed YRW Mb) and F43Y/F138W/P88W Mb (termed YW2 Mb) by introducing single or double Trp residues close to the heme active site [30,31,32]. Specifically, the triple mutant YW2 Mb exhibited considerably enhanced DyP activity, with the catalytic efficiency (*k*_cat_/*K*_m_) ~7-fold higher than that of *Vc*DyP, which is close to that of *Tfu*DyP [33,34].

With these achievements, we envisaged that the combination of not only the conserved His-Arg pair but also the electron-transfer Trp residues in Mb will confer a functional peroxidase. To test this hypothesis, we constructed a quadruple mutant F43Y/T67R/P88W/F138W Mb (termed YRW2 Mb, Figure 1C). As shown in this study, the quadruple mutant exhibits considerable peroxidase activity such as DyP activities toward a variety of synthetic dyes. Moreover, it is capable of lignin bioconversion, such as depolymerization of Kraft lignin and the dimer lignin model compound, guaiacylglycerol-β-guaiacyl ether (GGE).

## 2. Results and Discussion

### 2.1. Peroxidase Activity

The quadruple mutant YRW2 Mb was expressed and purified using the same procedure as that for WT Mb [35], which was further confirmed by ESI-MS analysis (Figure S1). We first investigated the H_2_O_2_-dependent peroxidase activity of YRW2 Mb in the oxidation of ABTS (Figure S2). The results showed that the enzyme exhibited a turnover number (*k*_cat_) of 40.6 ± 0.9 s^−1^ and a *K*_m_ of 5.5 ± 0.4 mM for H_2_O_2_. No obvious inhibition effect was observed for H_2_O_2_ at a concentration >20 mM, suggesting the tolerance of H_2_O_2_, which is similar to that of YW2 Mb, as reported in the previous study [31].

**Figure 2:**
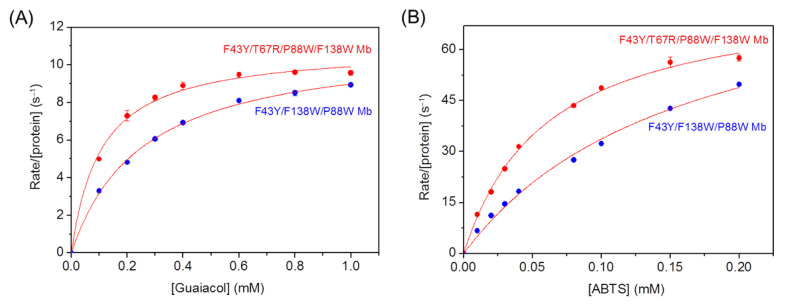
Steady-state rates of H_2_O_2_-dependent oxidation of guaiacol (**A**) and ABTS (**B**) catalyzed by F43Y/F138W/P88W Mb and F43Y/T67R/P88W/F138W Mb, with increasing the substrate concentrations. Reaction conditions: 2 μM protein, 20 mM H_2_O_2_, 50 mM potassium phosphate buffer at pH 7.0, 25 °C. The data were fitted to the Michaelis–Menten equation.

We then evaluated the peroxidase activity of the quadruple mutant YRW2 Mb and the triple mutant YW2 Mb, using both guaiacol and ABTS as the typical substrates under the steady-state conditions at a concentration of 20 mM H_2_O_2_. The obtained parameters are listed in Table 1, which are compared with those of WT Mb and mutants, as well as HRP. As shown in Figure 2 and Table 1 for the oxidation of guaiacol and ABTS at pH 7.0, 25 °C, although the quadruple mutant YRW2 Mb exhibited similar catalytic rates (*k*_cat_) to those of YW2 Mb under the same conditions, it showed largely decreased *K*_m_ values compared with the triple mutant. As a consequence, the catalytic efficiency of YRW2 Mb in oxidizing these two substrates is ~2.3 fold higher than those of YW2 Mb. It should be noted that although the contribution of R67 in *k*_cat_ could hardly be deduced between the quadruple and triple mutant, its role was indicated by the comparison of F43Y Mb and F43Y/T67R Mb (Table 1). Moreover, a distal Arg was demonstrated to be essential for regulating the structure and reactivity of artificial heme enzymes [36,37].

As further compared in Table 1 for guaiacol oxidation, YRW2 Mb showed an overall catalytic efficiency (*k*_cat_/*K*_m_ = 103,400 M^−1^s^−1^) ~4-fold, ~11.5-fold, ~26-fold, and ~940-fold higher than that of F43Y/T67R/F138W Mb (termed YRW Mb), F43Y/T67R Mb, F43Y Mb, and WT Mb, respectively, under the same conditions [32,38]. Moreover, the catalytic efficiency is much higher than that of natural lignin peroxidase (*k*_cat_/*K*_m_ = 72,000 M^−1^s^−1^) [39], as well as the most efficient HRP (Table 1) [40]. These results indicate that the combination of the quadruple mutations in Mb dramatically enhances the peroxidase activity, even exceeding that of the natural peroxidases.

For the oxidation of ABTS, the catalytic efficiency of the quadruple mutant is ~14-fold, ~37-fold, and ~287.5-fold higher than that of F43Y/T67R Mb, F43Y Mb, and WT Mb, respectively [32,38], which could be attributed to both the enhanced *k*_cat_ value and decreased *K*_m_ value. When compared to the triple mutant YRW Mb, YRW2 Mb exhibits both higher *k*_cat_ and *K*_m_ values, resulting in slightly lower catalytic efficiency. Moreover, although the quadruple mutant exhibits a slightly lower *k*_cat_ value compared to that of the triple mutant YW2 Mb, it shows a lower *K*_m_ value, resulting in ~2.3-fold higher catalytic efficiency. These observations suggest that the presence of the distal Arg67 and Trp88 close to the heme group may fine-tune both the protein reactivity and the binding of substrate. It is worth noting that due to the significant decrease in *K*_m_ value compared to that of the natural enzyme, the catalytic efficiency of the quadruple mutant is similar to those of HRP, as previously determined under the optimized conditions (pH 4.6–5.0) (Table 1) [40,41].

### 2.2. Dye-Decolorizing Peroxidase Activity

**Figure 3:**
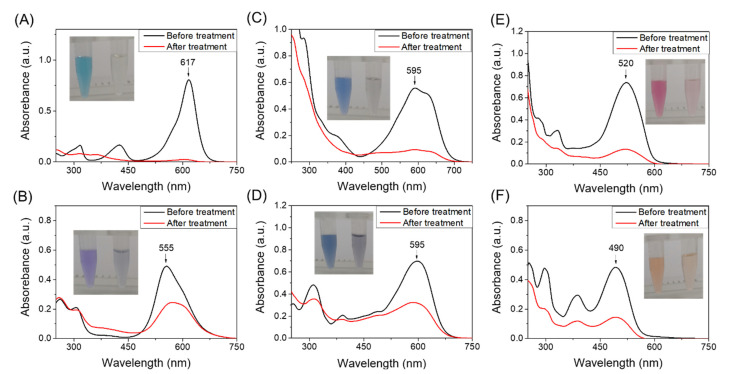
UV–vis absorption spectra of organic dyes before and after treatment by F43Y/T67R/P88W/F138W Mb (5 µM) in the presence of H_2_O_2_ (5 mM): (**A**) Malachite Green Oxalate (5 µM), (**B**) Brilliant blue R (20 µM), (**C**) Reactive blue 19 (60 µM), (**D**) Reactive black 5 (20 µM), (**E**) Amaranth (20 µM), and (**F**) Reactive orange 16 (20 µM).

We then evaluated the dye-decolorizing peroxidase activity of the quadruple mutant YRW2 Mb toward different organic dyes, including malachite green (MG), brilliant blue R (BBR), reactive blue 19 (RB19), reactive black 5 (RB5), amaranth (Ama), and reactive orange 16 (RO16). The experiments were performed on UV-vis spectrophotometer under optimal operational conditions (i.e., 50 mM potassium phosphate buffer, pH 7.0, with an addition of 5 mM H_2_O_2_) and the spectral changes were monitored after incubation for 0.5–1 h. As shown in Figure 3 and Table S1, the enzyme was able to decolorize all tested dyes. A remarkable dye-decolorizing effect was observed for the triphenylmethane dye, MG, after incubation for 1 h (~94%, Figure 3A) albeit with ~54% for BBR (Figure 3B). This decolorization effect is similar to that observed for the natural enzyme laccase in the decolorization of MG for 24 h (~90%) [42]. Moreover, for the decolorization of anthraquinone dyes such as RB19, the catalytic efficiency achieved ~85% after incubation for less than 10 s (Figure 3C), which suggests that YRW2 Mb is more reactive toward the decolorization of RB19.

Further study on the decolorization of double azo dyes, we found that the decolorization effect of YRW2 Mb on RB5 after incubation for 30 min (~54%, Figure 3D) was similar to BBR (Figure 3B). Meanwhile, a much higher decolorization effect was observed for the decolorization of single azo dyes, such as Ama (~82%, Figure 3E) and RO16 (~70%, Figure 3F), after incubation for 30 min under the same conditions. These observations agree with a previous report for a white rot bacterium, CBR43, which achieved the maximum decolorization effect for single azo dyes (i.e., 51–80% decolorization in 9 days) [18]. Therefore, these results suggest that the quadruple mutant YRW2 Mb has potential applications in the decolorization of various types of synthetic dyes.

It should be noted that some of the dyes show different protonation states at pH 7.0, which might affect the decolorization efficiency. It is different from that natural DyPs work at considerably low pH values (pH 3–4) [24], the enzymes designed in Mb work well at pH 6–7, which is thus more environmentally friendly [30,31]. Moreover, these organic dyes have different redox potentials, which may also affect the decolorization activity of the designed heme enzymes [17]. Therefore, further investigation of the relationship should be a future direction.

### 2.3. Bioconversion of Kraft Lignin

**Figure 4:**
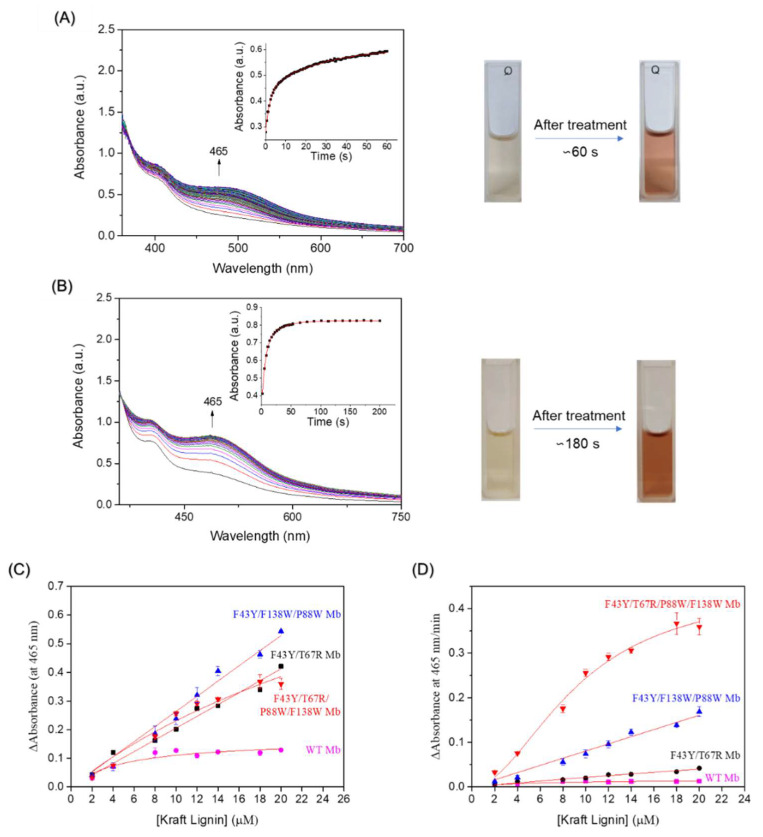
Kinetic studies of Kraft lignin (14 µM) oxidation in the presence of H_2_O_2_ (2.0 mM) catalyzed by (**A**) F43Y/T67R/P88W/F138W Mb (2 µM) and (**B**) F43Y/F138W/P88W Mb (2 µM), respectively. Time-dependent changes of the absorbance at 465 nm were shown as insets and were fitted to the double-exponential decay function. The color changes upon reaction were shown in the right column. (**C**) The changes of absorbance at 465 nm after reacting for 1–10 min. (**D**) steady-state rates of oxidation with different concentrations of Kraft lignin, as catalyzed by F43Y/T67R/P88W/F138W Mb, F43Y/F138W/P88W Mb, F43Y/T67R Mb, and WT Mb, respectively. The plots were fitted to the Hill and Michaelis-Menten equations, respectively. The error bars in C and D were generated from triplicate experiments.

Given the complex structure of lignin, there are different options to evaluate the enzyme activity in reaction with lignin. We carried out the experiments on kraft lignin and studied the kinetics of kraft lignin oxidation by monitoring the change of absorbance at 465 nm. As shown in Figure 4A, the oxidation rate catalyzed by YRW2 Mb increased at first ~20 s and reached a plateau after ~60 s, with an obvious rate constant (*k*_obs_) of 0.451 ± 0.013 s^−1^. Control study using YW2 Mb showed that the reaction rate was considerably slower (*k*_obs_ = 0.162 ± 0.003 s^−1^, Figure 4B), reaching a plateau after ~100 s. The visual appearances upon the oxidation of Kraft lignin were shown in Figure 4A,B (right column), which showed red for the solutions after the treatment of Kraft lignin.

**Figure 5:**
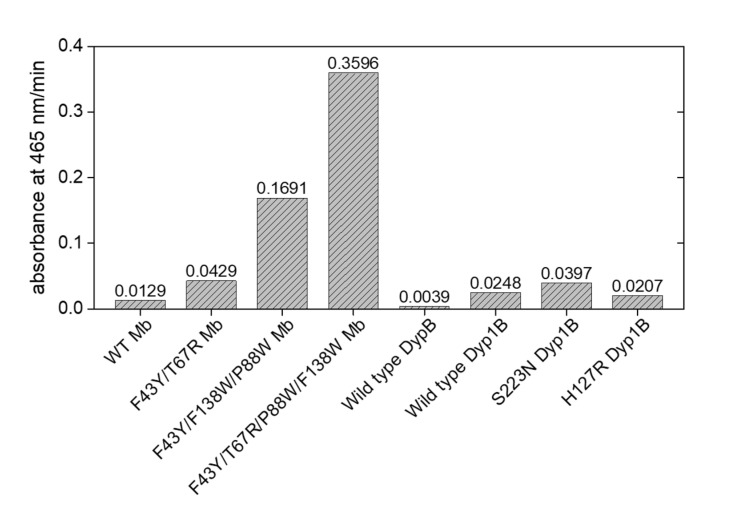
The enzymatic activities for Mb and its mutants in the oxidation of Kraft lignin, with the native Dyp and its mutants shown for comparison.

We also studied the dependence of Kraft lignin concentration on enzymatic activity. As shown in Figure 4C, the absorbance at 465 nm increased with the increase of lignin concentration for the reaction catalyzed by YRW2 Mb. Similar results were observed for the double and triple mutants, F43Y/T67R Mb and YW2 Mb. Further analysis showed that, although the change in absorbance of Kraft lignin catalyzed by YRW2 Mb was not as high as that of YW2 Mb, the catalytic rate of the quadruple mutant (0.3596 a.u/min) was ~2-fold higher compared to that of YW2 Mb (0.1691 a.u./min) (Figure 4D). As compared in Figure 5, the activity is ~8-fold and ~28-fold higher than F43Y/T67R Mb (0.0429 a.u./min) and WT Mb (0.0129 a.u./min), respectively [27]. Moreover, the enzymatic activity with 20 µM Kraft lignin was ~9–17-fold higher than those reported recently for the natural peroxidase Dyp1B (0.0248 a.u./min) or its mutants S223N (0.0397 a.u./min) and H127R (0.0207 a.u./min) (Figure 5) [43]. These results suggest that YRW2 Mb was efficient in catalyzing the bioconversion of Kraft lignin, although it was difficult to analyze the products due to the complexity.

### 2.4. Bioconversion of Model Compound GGE

**Figure 6:**
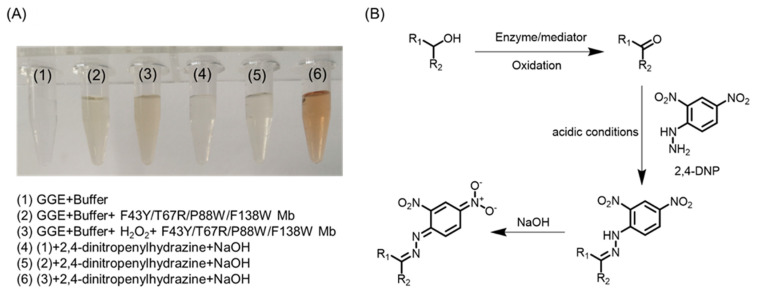
(**A**) Visual appearance of GGE (2.0 mM) oxidation in the absence (1) and presence (2–3) of F43Y/T67R/P88W/F138W Mb (5 µM) and H_2_O_2_ (2.0 mM) (3) for 10 min. After the reactions (1), (2), and (3) mixed with 2,4-dinitropenylhydrazine (2,4-DNP, 0.3 mM) for 15 min, the visual appearance of reactions in the presence (4–6) of NaOH (0.5 mM). (**B**) Reaction overview of 2,4-DNP with carbonyl groups [44].

To identify possible products of lignin bioconversion and provide information for the reaction mechanism, we chose to evaluate the oxidation of the lignin model compound, guaiacylglycerol-β-guaiacyl ether (GGE), a phenolic type model compound containing the β-O-4 bond that constitutes 50–70% of intersubunit bonds in Kraft lignin. At first, we monitored the visual appearance during the oxidation of GGE catalyzed by the quadruple mutant (Figure 6A). The results of an addition-elimination reaction with 2,4-dinitropenylhydrazine (2,4-DNP) indicated that an alcoholic group in GGE was oxidized to a ketone or aldehyde. As shown in Figure 6B, when GGE was oxidized to produce a carbonyl group, it spontaneously reacted with 2,4-DNP in acidic conditions, producing a dinitrophenylhydrazone derivative, which appeared red after reacting with NaOH [44].

After the assays, the resulting solutions appeared yellow in the treatment of GGE by YRW2 Mb in the presence of H_2_O_2_ (Figure 6, entry 3), which was not observed for buffer solution containing GGE in the absence or presence of the enzyme (Figure 6, entries 1–2). After the addition of 2,4-DNP and NaOH to the buffer containing GGE in the absence or presence of H_2_O_2_ and the enzyme, the results showed that in the presence of H_2_O_2_, the product of GGE catalyzed by enzyme reacted with 2,4-DNP and showed red for the solution (Figure 6, entry 6). Therefore, this colorimetric assay suggests that GGE oxidation was coupled to its depolymerization, and the products were further analyzed in the following sections.

### 2.5. HPLC and ESI-MS Analysis of the Products

**Figure 7:**
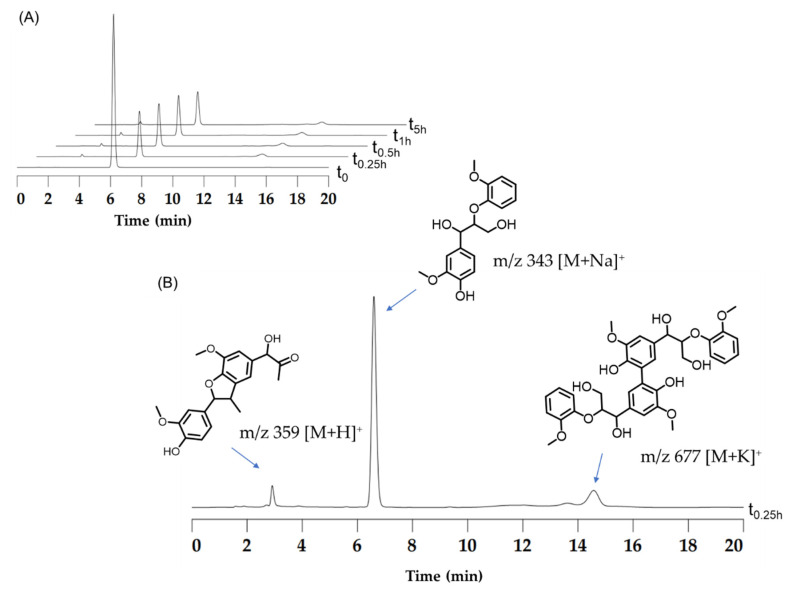
(**A**) HPLC traces as monitored at 280 nm, showing the oxidation of 2.5 mM of GGE catalyzed by F43Y/T67R/P88W/F138W Mb (5 μM) in the presence of H_2_O_2_ (2 mM) in potassium phosphate buffer (pH 7.0). (**B**) Analysis of the products by mass spectrometry in positive mode, showing *m/z* 359 [M+H]^+^ and 677 [M+K]^+^.

To analyze the oxidation products of the dimeric lignin model GGE, we performed both HPLC and mass spectrometry analysis. As shown in Figure 7A for HPLC analysis, the oxidation of GGE catalyzed by YRW2 Mb resulted in a ~65% conversion after reacting for 0.5 h at pH 7.0, and the yield remained similar up to 5 h. Previous studies showed that other natural or artificially modified enzymes can also only oxidize GGE to 60–70%, such as lignin peroxidase from *P*. *eryngii* and the *Pp*DyP 6E10 variant from *P*. *putida* MET94 [21,45]. Note that the signal of the remaining GGE was observed at 6.607 min in the HPLC trace (Figure 7B).

**Figure 8:**
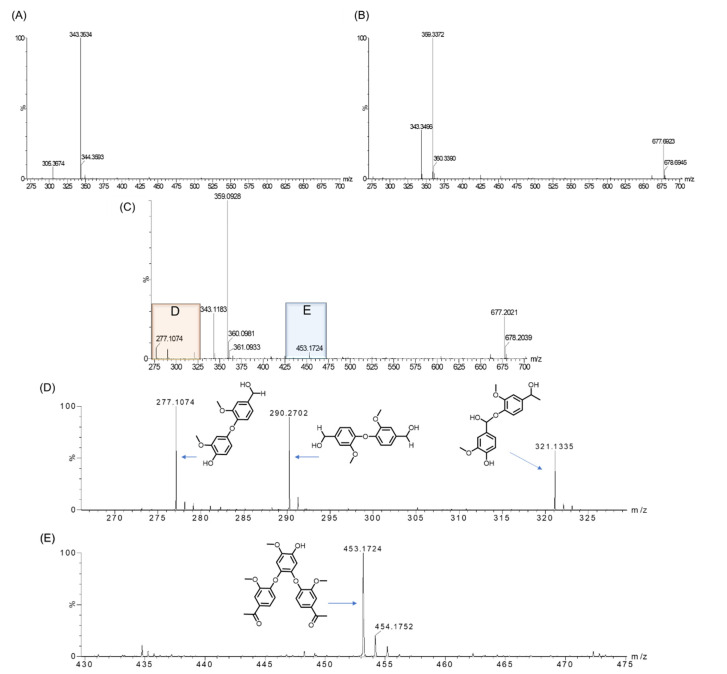
(**A**) ESI-MS spectra of the GGE with the molecular weight of 343 Da. (**B**) After reacting for 0.25 h, two major products were detected with molecular weights of 359 and 677 Da, respectively. (**C**) After reacting for 1 h, four more products were detected, with molecular weights of 277, 290, 321 (**D**), and 453 Da (**E**).

The corresponding ESI-MS results revealed the product peaks in a range of *m/z* 260–700, which were higher than the molecular weight of the starting GGE (320 Da, observed, 343 Da, [M + Na]^+^) (Figure 8A). After reacting for 15 min, we detected the formation of two products by HPLC with retention times (RT) of 2.919 min and 14.554 min, respectively (Figure 7B). Moreover, ESI-MS analysis showed that the molecular weights of the two products were *m/z* 359 and 677 Da (Figure 8B), which are consistent with the molecular formulas of C_20_H_22_O_6_ and C_34_H_38_O_12_, respectively. Note that the product with a retention time of 14.554 min was a dimer of GGE, which is similar to the HPLC-MS result of the oxidized GGE product as catalyzed by *Pp*DyP 6E10 variant [21].

Moreover, despite only two major product peaks in the HPLC trace after 0.25 h (Figure 7B), ESI-MS analysis showed that four product peaks appeared in the D and E regions after reacting for 1 h (Figure 8C). This might be due to the low content of the product that was below the detection limit of HPLC. As shown in Figure 8D,E for these two regions, these mass signals showed *m/z* 277 [M+H]^+^, 290 [M]^+^, 321 [M+H]^+^, and 453 [M+H]^+^, which are consistent with the molecular formulas of C_15_H_16_O_5_, C_16_H_18_O_5_, C_17_H_20_O_6_, and C_25_H_24_O_8_, respectively. These results indicate that as catalyzed by YRW2 Mb, the main GGE degradation patterns were caused by the oxidative cleavage of the C-C or C-O bond, which produced small molecules such as those detected with molecular weights of 277, 290, 321, and 453 Da. These products may further undergo polymerization, as proposed in the following section.

### 2.6. Proposed Mechanism for GGE Bioconversion

**Figure 9:**
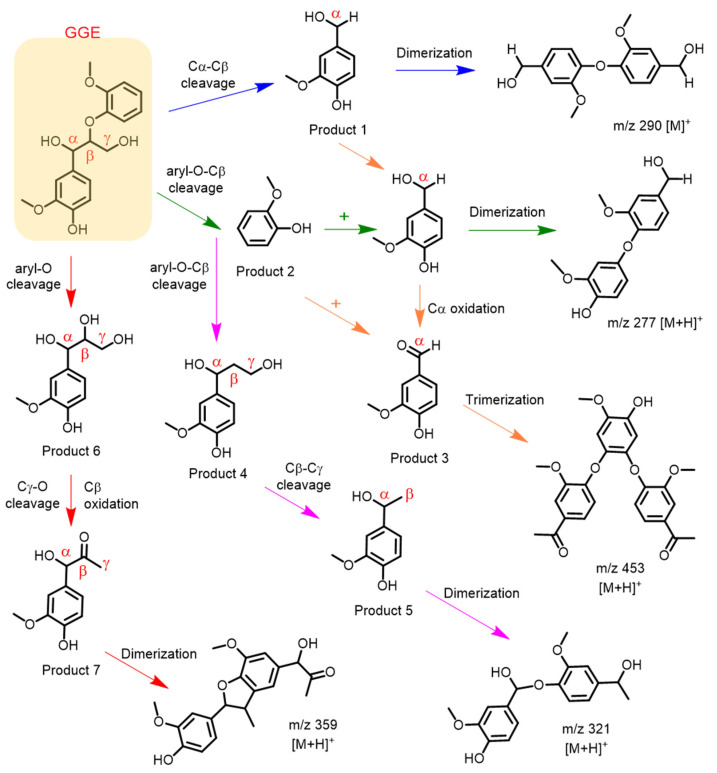
Depolymerization and polymerization of the GGE catalyzed by F43Y/F138W/P88W/T67R Mb. Product **1** is 4-(hydroxymethyl)-2-methoxyphenol, product **2** is guaiacol, product **3** is vanillin, product **4** is 1-(4-hydroxy-3-methoxyphenyl)propane-1,3-diol, product **5** is 4-(1-hydroxyethyl)- 2-methoxyphenol, product **6** is 1-(4-hydroxy-3-methoxyphenyl)propane-1,2,3-triol, and product **7** is 1-hydroxy-1-(4-hydroxy-3-methoxyphenyl)propan-2-one, respectively.

Based on the above HPLC and ESI-MS results, we proposed a generation route of the reaction products, which may involve the generation of reactive radical species upon the activation of H_2_O_2_ by the heme enzymes [13,19,30,46,47], resulting in various types of bond cleavages. As shown in Figure 9, for the oxidative cleavage, the monomers can hardly exist alone, and they might be recombined into new species. For example, by Cα-Cβ cleavage of GGE, product 1 (4-(hydroxymethyl)-2-methoxyphenol) will be generated, which further forms a dimer, matching the expected molecular formulas of C_16_H_18_O_5_, with a molecular weight of 290 [M]^+^. The reaction will also produce product 2 (guaiacol) by the cleavage of aryl-O-Cβ bond in GGE. Note that products 1 and 2 may be coupled to form a heterodimer, with an expected molecular formula of C_15_H_16_O_5_ and *m/z* 277 [M+H]^+^. A previous study showed that the degradation of GGE by *Tc*DyP also produced the product of guaiacol, whereas it ultimately formed guaiacol pentamer [24].

Moreover, after the Cα-Cβ or aryl-Cα cleavage of GGE, the reaction may continue to generate product **3** (vanillin) by oxidation of Cα. Then products **2** and **3** may form a heterotrimer linked by C-O-C bonds, generating the observed molecule of C_25_H_24_O_8_ with *m/z* 453 [M+H]^+^. It should be noted that the detection of vanillin from a lignin dimer was reported previously for *R*. jostii DypB, which revealed that the oxidative cleavage of Cα-Cβ occurred, although dimerization via the coupling of phenoxy radicals also occurred [48].

As shown in Figure 9, upon the aryl-O-Cβ cleavage of GGE, in addition to product **2**, the depolymerization product **4**, (1-(4-hydroxy-3-methoxyphenyl)propane-1,3-diol), may also be generated. It will be further cleaved by Cβ-Cγ to produce product **5**, (4-(1-hydroxyethyl)-2-methoxyphenol). Product **5** may undergo dimerization to form a dimer, with the molecular formula of C_17_H_20_O_6_. Alternatively, GGE may be cleaved by the aryl-O bond, producing product **6**, (1-(4-hydroxy-3-methoxyphenyl)propane- 1,2,3-triol). Furthermore, it will produce product **7**, (1-hydroxy-1-(4-hydroxy-3- methoxyphenyl)propan-2-one), by both Cγ-O cleavage and Cβ oxidation of product **6**, which will also form a dimer, with a molecular weight corresponding to the molecular formula of C_20_H_22_O_6_ (*m/z* 359) that has an RT of 2.919 min. Based on these proposed degradation pathways for the model compound GGE, we can infer the possibility that YRW2 Mb could attack at the corresponding catalytic cracking sites of Kraft lignin in bioconversion.

## 3. Materials and Methods

### 3.1. Materials

Malachite green (MG), Brilliant blue R (BBR), Reactive blue 19 (RB19), Reactive black 5 (RB5), amaranth (Ama), and Reactive orange 16 (RO16) were bought from Adamas-beta (Shanghai, China). The peroxidase substrates, guaiacol, and 2,2′-azino-bis (3-ethylbenzothiazoline-6-sulfonic acid) diammonium salt (ABTS) were bought from Aladdin Industrial Corporation (Shanghai, China). Guaiacylglycerol-β-guaiacyl ether (GGE) was purchased from Shanghai Macklin Biochemical Co., Ltd. (Shanghai, China). 2,4-dinitrophenylhydrazine (2,4-DNP) were bought from Adamas-beta. Lignin and alkali (Lignin, Kraft) were bought from Sigma-Aldrich (Saint Louis, MO, USA). All other reagents were of analytical grade.

### 3.2. Protein Expression and Purification

WT sperm whale Mb was expressed in the host *E. coli* BL21(DE3) cells transformed with the pMbt7–7-Mb plasmid [49]. The gene of F43Y/T67R/P88W/F138W Mb (YRW2 Mb) was constructed by the QuikChange Site-Directed Mutagenesis Kit (Stratagene, Inc. Santa Clara, CA, USA) using the WT Mb gene as a template, and the mutations were identified by DNA sequencing. The quadruple mutant YRW2 Mb was expressed and purified using the same procedure as previously reported [31], which was further confirmed by ESI-MS analysis (Figure S1). The H_2_O_2_-dependent of the peroxidase activities were performed under similar conditions by varying the concentrations of H_2_O_2_ (0~25 mM) (Figure S2).

### 3.3. Peroxidase Activity Assay

The kinetic parameters for the peroxidase activity of YRW2 Mb were investigated on a dual mixing stopped-flow spectrophotometer (SF-61DX2 Hi-Tech KinetAsystTM) (Hi-Tech Scientific, Bradford-on-Avon, UK) using guaiacol (0–1.0 mM, ε_470nm_ = 26.6 mM^−1^ cm^−1^) or ABTS (0–0.2 mM, ε_660nm_ = 14 mM^−1^ cm^−1^) as the substrate, in 50 mM potassium phosphate buffer, pH 7.0, 25 °C, to which H_2_O_2_ (20 mM) was added. Control experiments were performed with the triple mutant F43Y/F138W/P88W Mb (YW2 Mb) under the same conditions.

### 3.4. Dye-Decolorizing Peroxidase Activity Assay

The synthetic dyes MG, BBR, RB19, RB5, Ama, and RO16 were used to evaluate the decolorization capability of YRW2 Mb. The reaction mixture contained 5–60 μM dye and 5 μM YRW2 Mb, to which 5 mM H_2_O_2_ was added (final volume, 2 mL), in 50 mM potassium phosphate buffer, pH 7.0. After incubation for 0.5–1 h at room temperature on a rotary wheel, the absorbance spectra were recorded from 250 to 750 nm on a Lambda 365 spectrophotometer (PerkinElmer, Inc., Waltham, MA, USA). The control experiments were performed under the same conditions without the addition of the enzyme. The dye decolorization was expressed in percentage as follows [18,50]:

Dec-l = [(Abst0 − Abst1)]/Abst0 × 100, where Dec-l is the decolorization level (%), and Abst0 and Abst1 are the absorbance values before and after treatment, recorded at 617, 555, 595, 595, 520, and 490 nm for MG, BBR, RB19, RB5, Ama, and RO16 dyes, respectively.

### 3.5. Kinetic Analysis of Alkali Kraft Lignin

Kraft lignin (10 mg) was dissolved in DMSO (1 mL). 14 μM of the sample, 2 μM YW2 Mb or YRW2 Mb, and 2 mM H_2_O_2_ were mixed in potassium phosphate buffer (pH 7.0, 50 mM) containing DMSO (5%, *v*/*v*), with a final volume of 2 mL. The spectrum was monitored for 1–10 min at 465 nm on an Agilent 8453 diode array spectrometer (Agilent Technologies, Inc., Santa Clara, CA, USA) or a stopped-flow spectrophotometer. Substrate concentrations were then varied for Kraft lignin (final concentration, 2–20 μM), with the total volume kept at 2 mL. The concentration was calculated using an average molecular mass of 10,000 Da.

### 3.6. Assay for Ketone Products

Upon oxidation of GGE catalyzed by YRW2 Mb, for detecting any released aldehydes or ketones molecules, an assay was used based on the reaction of aldehydes with 2,4-DNP, which forms a colored hydrazone product [44]. Briefly, 2.0 mM GGE dissolved in 1/1 ethanol/H_2_O was added to the potassium phosphate buffer (2 mL, 50 mM, pH 7.0), followed by the addition of YRW2 Mb and H_2_O_2_ (2 mM). The resulting solution was incubated at 25 °C for 1 h. Then, 200 μL of the solution was mixed with 300 μL HCl (100 mM) followed by the addition of 500 μL of 2,4-DNP (1 mM dissolved in 100 mM HCl). The mixture was incubated at 25 °C for 5 min and then 1000 μL NaOH (100 mM) was added, as described previously [51].

### 3.7. HPLC and Mass Spectrometry

A 20 mM GGE solution was prepared by dissolving 25 mg of the substrate in 1 mL ethanol and diluted to a final volume of 2 mL with water. The assay mixture containing 50 mM potassium phosphate buffer, pH 7.0, 2.5 mM GGE, 2 mM H_2_O_2_, and 5 μM YRW2 Mb was incubated at 25 °C (final volume, 2.0 mL). At fixed times, 300 μL of the reaction solution was added with 100 μL of acetonitrile, followed by passing the 0.22 mm filter membrane. 20 μL of the filtrate was analyzed by HPLC analysis using a Shim-pack GIST 5 μm C18 column (150/4.6 mm), detection set at 280 nm. Elution gradient: 0–20 min, 30% acetonitrile/70% H_2_O. The retention times (RT) of GGE were 6.607 min. The remaining filtrate was carried out on G2-XS QTOF mass spectrometry (Waters, Milford, MA, USA), and the samples were transferred into the mass spectrometer chamber for measurement under a positive mode.

## 4. Conclusions

Inspired by the key structural feature of natural heme enzymes, in this study we rationally designed an efficient peroxidase using Mb as a protein model, by introducing both the conserved His-Arg pair for catalysis and the Tyr/Trp for electron transfer in the heme active site. Kinetic studies showed that the designed quadruple mutant F43Y/T67R/P88W/F138W Mb exhibits remarkable peroxidase activity, with the catalytic efficiency similar to that of the most efficient natural enzyme HRP. The quadruple mutant is also able to decolorize multiple types of synthetic textile dyes, including triphenylmethane-type dyes, azo-type dyes, and anthraquinone-type dyes. Moreover, this enzyme exhibits a potential for bioconversion of lignin, such as Kraft lignin and the model lignin dimer GGE, representative of the main-chain linkages in lignin. By analysis using HPLC and ESI-MS, we identified several bioconversion products of GGE, as produced via Cα-Cβ, aryl-Cα, aryl-O-Cβ, Cβ-Cγ, or Cβ-O cleavage, followed by dimerization or trimerization, which illustrates the mechanism for the bioconversion of lignin. This study indicates that the rationally designed F43Y/T67R/P88W/F138W Mb is an efficient multi-functional peroxidase, which could thus be exploited for the decolorization of textile wastewater contaminated with various dyes, as well as for the bioconversion of lignin from the industry to produce more value-added products.

## Figures and Tables

**Table 1:** Kinetic parameters for H_2_O_2_-dependent oxidation of guaiacol and ABTS catalyzed by Mb and its mutants. Measurements were performed in 50 mM potassium phosphate buffer at pH 7.0, 25 °C. The parameters of natural lignin peroxidase and HRP are shown for comparison.

Enzyme	*k*_cat_ (s^−1^)	*K*_m_ (mM/μM)	*k*_cat_ /*K*_m_ (M^−1^s^−1^)
Guaiacol (*K*_m_, mM)			
WT Mb [38]	0.4 ± 0.1	3.53 ± 0.05	110
F43Y Mb [38]	10.7 ± 0.4	2.67 ± 0.21	4000
F43Y/T67R Mb [32]	23.5 ± 0.3	2.61 ± 0.06	9000
F43Y/T67R/F138W Mb [32]	27.7 ± 0.8	0.79 ± 0.07	35,000
F43Y/F138W/P88W Mb	11.3 ± 0.2	0.25 ± 0.01	44,380
F43Y/T67R/P88W/F138W Mb	11.0 ± 0.2	0.11 ± 0.01	103,400
Lignin peroxidase [39]	7.7 ± 0.0	0.16 ± 0.00	48,000
HPR [40]	420 ± 40.0	5.8 ± 0.70	72,000
ABTS (*K*_m_, μM)			
WT Mb [38]	0.55 ± 0.02	124 ± 15	4440
F43Y Mb [38]	12.0 ± 0.68	351 ± 40	34,190
F43Y/T67R Mb [32]	50.8 ± 3.6	567 ± 68	89,600
F43Y/T67R/F138W Mb [32]	31.5 ± 0.6	16 ± 2	1,970,000
F43Y/F138W/P88W Mb	88.5 ± 6.64	162 ± 21	545,050
F43Y/T67R/P88W/F138W Mb	76.8 ± 1.89	60 ± 4	1,276,570
HRP [40]	340 ± 60	430 ± 20	800,000
HRP [41]	332 ± 18	233 ± 21	1,420,000

## Data Availability

The datasets for this manuscript can be obtained from the corresponding author upon reasonable request.

## Supplementary Materials

The following are available online at https://www.mdpi.com/article/10.3390/ijms23010413/s1.

## References

[1] Ali S., Peter A.P., Chew K.W., Munawaroh H.S.H., Show P.L. (2021). Resource recovery from industrial effluents through the cultivation of microalgae: A review. Bioresour. Technol..

[2] Andersson E., Dernegard H., Wallen M., Thollander P. (2021). Decarbonization of industry: Implementation of energy performance indicators for successful energy management practices in kraft pulp mills. Energy Rep..

[3] Sosa-Martinez J.D., Balagurusamy N., Montanez J., Peralta R.A., Moreira R.D.P.M., Bracht A., Peralta R.M., Morales-Oyervides L. (2020). Synthetic dyes biodegradation by fungal ligninolytic enzymes: Process optimization, metabolites evaluation and toxicity assessment. J. Hazard. Mater..

[4] Singh A.K., Bilal M., Iqbal H.M.N., Meyer A.S., Raj A. (2021). Bioremediation of lignin derivatives and phenolics in wastewater with lignin modifying enzymes: Status, opportunities and challenges. Sci. Total Environ..

[5] Gogate P.R., Pandit A.B. (2004). A review of imperative technologies for wastewater treatment I: Oxidation technologies at ambient conditions. Adv. Environ. Res..

[6] Brebu M., Vasile C. (2010). Thermal degradation of lignin—A Review. Cellul. Chem. Technol..

[7] Zhou X.F. (2014). Conversion of kraft lignin under hydrothermal conditions. Bioresour. Technol..

[8] Lin Y.-W. (2017). Rational design of metalloenzymes: From single to multiple active sites. Coord. Chem. Rev..

[9] Yin L.-L., Yuan H., Liu C., He B., Gao S.-Q., Wen G.-B., Tan X., Lin Y.-W. (2018). A Rationally Designed Myoglobin Exhibits a Catalytic Dehalogenation Efficiency More than 1000-Fold That of a Native Dehaloperoxidase. ACS Catal..

[10] Nastri F., D’Alonzo D., Leone L., Zambrano G., Pavone V., Lombardi A. (2019). Engineering Metalloprotein Functions in Designed and Native Scaffolds. Trends Biochem. Sci..

[11] Lin Y.-W. (2020). Rational design of heme enzymes for biodegradation of pollutants toward a green future. Biotechnol. Appl. Biochem..

[12] Lin Y.-W. (2021). Biodegradation of aromatic pollutants by metalloenzymes: A structural-functional-environmental perspective. Coord. Chem. Rev..

[13] Chen S.-F., Liu X.-C., Xu J.-K., Li L., Lang J.-J., Wen G.-B., Lin Y.-W. (2021). Conversion of Human Neuroglobin into a Multifunctional Peroxidase by Rational Design. Inorg. Chem..

[14] Cajnko M.M., Oblak J., Grilc M., Likozar B. (2021). Enzymatic bioconversion process of lignin: Mechanisms, reactions and kinetics. Bioresour. Technol..

[15] Chatha S.A.S., Asgher M., Iqbal H.M.N. (2017). Enzyme-based solutions for textile processing and dye contaminant biodegradation-a review. Environ. Sci. Pollut. Res. Int..

[16] Xu H., Guo M.Y., Gao Y.H., Bai X.H., Zhou X.W. (2017). Expression and characteristics of manganese peroxidase from Ganoderma lucidum in Pichia pastoris and its application in the degradation of four dyes and phenol. BMC Biotechnol..

[17] Jenkins J.M.X., Noble C.E.M., Grayson K.J., Mulholland A.J., Anderson J.L.R. (2021). Substrate promiscuity of a de novo designed peroxidase. J. Inorg. Biochem..

[18] Yang S.O., Sodaneath H., Lee J.I., Jung H., Choi J.H., Ryu H.W., Cho K.S. (2017). Decolorization of acid, disperse and reactive dyes by Trametes versicolor CBR43. J. Environ. Sci. Health A Tox. Hazard. Subst. Environ. Eng..

[19] Chan J.C., Paice M., Zhang X. (2019). Enzymatic Oxidation of Lignin: Challenges and Barriers Toward Practical Applications. ChemCatChem.

[20] Colpa D.I., Fraaije M.W., van Bloois E. (2014). DyP-type peroxidases: A promising and versatile class of enzymes. J. Ind. Microbiol. Biot..

[21] Brissos V., Tavares D., Sousa A.C., Robalo M.P., Martins L.O. (2017). Engineering a Bacterial DyP-Type Peroxidase for Enhanced Oxidation of Lignin-Related Phenolics at Alkaline pH. ACS Catal..

[22] Rahmanpour R., Rea D., Jamshidi S., Fulop V., Bugg T.D.H. (2016). Structure of Thermobifida fusca DyP-type peroxidase and activity towards Kraft lignin and lignin model compounds. Arch. Biochem. Biophys..

[23] Chaplin A.K., Wilson M.T., Worrall J.A.R. (2017). Kinetic characterisation of a dye decolourising peroxidase from Streptomyces lividans: New insight into the mechanism of anthraquinone dye decolourisation. Dalton. Trans..

[24] Chen C., Shrestha R., Jia K., Gao P.F., Geisbrecht B.V., Bossmann S.H., Shi J., Li P. (2015). Characterization of Dye-decolorizing Peroxidase (DyP) from Thermomonospora curvata Reveals Unique Catalytic Properties of A-type DyPs. J. Biol. Chem..

[25] Yan D.J., Li W., Xiang Y., Wen G.B., Lin Y.W., Tan X.S. (2015). A Novel Tyrosine-Heme C-O Covalent Linkage in F43Y Myoglobin: A New Post-translational Modification of Heme Proteins. Chembiochem.

[26] Liu C., Yuan H., Liao F., Wei C.W., Du K.J., Gao S.Q., Tan X., Lin Y.W. (2019). Unique Tyr-heme double cross-links in F43Y/T67R myoglobin: An artificial enzyme with a peroxidase activity comparable to that of native peroxidases. Chem. Commun..

[27] Guo W.-J., Xu J.-K., Liu J.-J., Lang J.-J., Gao S.-Q., Wen G.-B., Lin Y.-W. (2021). Biotransformation of Lignin by an Artificial Heme Enzyme Designed in Myoglobin With a Covalently Linked Heme Group. Front. Bioeng. Biotechnol..

[28] Linde D., Pogni R., Canellas M., Lucas F., Guallar V., Baratto M.C., Sinicropi A., Saez-Jimenez V., Coscolin C., Romero A. (2015). Catalytic surface radical in dye-decolorizing peroxidase: A computational, spectroscopic and site-directed mutagenesis study. Biochem. J..

[29] Shrestha R., Chen X.J., Ramyar K.X., Hayati Z., Carlson E.A., Bossmann S.H., Song L.K., Geisbrecht B.V., Li P. (2016). Identification of Surface-Exposed Protein Radicals and A Substrate Oxidation Site in A-Class Dye-Decolorizing Peroxidase from Thermomonospora curvata. Acs Catal..

[30] Li L.L., Yuan H., Liao F., He B., Gao S.Q., Wen G.B., Tan X., Lin Y.W. (2017). Rational design of artificial dye-decolorizing peroxidases using myoglobin by engineering Tyr/Trp in the heme center. Dalton. Trans..

[31] Zhang P., Xu J., Wang X.-J., He B., Gao S.-Q., Lin Y.-W. (2019). The Third Generation of Artificial Dye-Decolorizing Peroxidase Rationally Designed in Myoglobin. ACS Catal..

[32] Liao F., Xu J.K., Luo J., Gao S.Q., Wang X.J., Lin Y.W. (2020). Bioinspired design of an artificial peroxidase: Introducing key residues of native peroxidases into F43Y myoglobin with a Tyr-heme cross-link. Dalton. Trans..

[33] van Bloois E., Pazmino D.E.T., Winter R.T., Fraaije M.W. (2010). A robust and extracellular heme-containing peroxidase from Thermobifida fusca as prototype of a bacterial peroxidase superfamily. Appl. Microbiol. Biot..

[34] Uchida T., Sasaki M., Tanaka Y., Ishimorit K. (2015). A Dye-Decolorizing Peroxidase from Vibrio cholerae. Biochemistry.

[35] Urayama P., Phillips G.N., Gruner S.M. (2002). Probing Substates in Sperm Whale Myoglobin Using High-Pressure Crystallography. Structure.

[36] Maglio O., Chino M., Vicari C., Pavone V., Louro R.O., Lombardi A. (2021). Histidine orientation in artificial peroxidase regioisomers as determined by paramagnetic NMR shifts. Chem. Commun..

[37] Leone L., D’Alonzo D., Maglio O., Pavone V., Nastri F., Lombardi A. (2021). Highly Selective Indole Oxidation Catalyzed by a Mn-Containing Artificial Mini-Enzyme. ACS Catal..

[38] Liao F., He B., Du K.-J., Gao S.-Q., Wen G.-B., Lin Y.-W. (2016). Enhanced Dehaloperoxidase Activity of F43Y Myoglobin with a Novel Thyrosine–Heme Crosslink. Chem. Lett..

[39] Koduri R.S., Tien M. (1995). Oxidation of guaiacol by lignin peroxidase. Role of veratryl alcohol. J. Biol. Chem..

[40] Savenkova M.I., Kuo J.M., Ortiz de Montellano P.R. (1998). Improvement of Peroxygenase Activity by Relocation of a Catalytic Histidine within the Active Site of Horseradish Peroxidase. Biochemistry.

[41] Fruk L., Müller J., Niemeyer C.M. (2006). Kinetic analysis of semisynthetic peroxidase enzymes containing a covalent DNA-heme adduct as the cofactor. Chemistry.

[42] Sinirlioglu Z.A., Sinirlioglu D., Akbas F. (2013). Preparation and characterization of stable cross-linked enzyme aggregates of novel laccase enzyme from Shewanella putrefaciens and using malachite green decolorization. Bioresour. Technol..

[43] Rahman Pour R., Ehibhatiomhan A., Huang Y., Ashley B., Rashid G.M., Mendel-Williams S., Bugg T.D.H. (2019). Protein engineering of Pseudomonas fluorescens peroxidase Dyp1B for oxidation of phenolic and polymeric lignin substrates. Enzym. Microb. Technol..

[44] Tonin F., Vignali E., Pollegioni L., D’Arrigo P., Rosini E. (2017). A novel, simple screening method for investigating the properties of lignin oxidative activity. Enzym. Microb. Technol..

[45] Caramelo L., Martinez M.J., Martinez A.T. (1999). A Search for Ligninolytic Peroxidases in the Fungus Pleurotus eryngii Involving α-Keto-γ-Thiomethylbutyric Acid and Lignin Model Dimers. Appl. Environ. Microbiol..

[46] Xu L., Sun J., Qaria M.A., Gao L., Zhu D. (2021). Dye Decoloring Peroxidase Structure, Catalytic Properties and Applications: Current Advancement and Futurity. Catalysts.

[47] Sun L.-J., Yuan H., Xu J.-K., Luo J., Lang J.-J., Wen G.-B., Tan X., Lin Y.-W. (2021). Phenoxazinone Synthase-like Activity of Rationally Designed Heme Enzymes Based on Myoglobin. Biochemistry.

[48] Ahmad M., Roberts J.N., Hardiman E.M., Singh R., Eltis L.D., Bugg T.D. (2011). Identification of DypB from Rhodococcus jostii RHA1 as a Lignin Peroxidase. Biochemistry.

[49] Springer B.A., Sligar S.G. (1987). High-level expression of sperm whale myoglobin in Escherichia coli. Proc. Natl. Acad. Sci. USA.

[50] Bilal M., Iqbal H.M.N., Hu H., Wang W., Zhang X. (2017). Enhanced bio-catalytic performance and dye degradation potential of chitosan-encapsulated horseradish peroxidase in a packed bed reactor system. Sci. Total Environ..

[51] Vignali E., Tonin F., Pollegioni L., Rosini E. (2018). Characterization and use of a bacterial lignin peroxidase with an improved manganese-oxidative activity. Appl. Microbiol. Biotechnol..

