# Supplementary material for: Design and Engineering of an Efficient Peroxidase Using Myoglobin for Dye Decolorization and Lignin Bioconversion
Source: Int J Mol Sci. 2021 Dec 30;23(1):413. doi: 10.3390/ijms23010413 (PMC8745427; doi:10.3390/ijms23010413)
Supplement: Supplementary file 1 [file ijms-23-00413-s001.zip › ijms-1505558-supplementary.pdf]

(A)

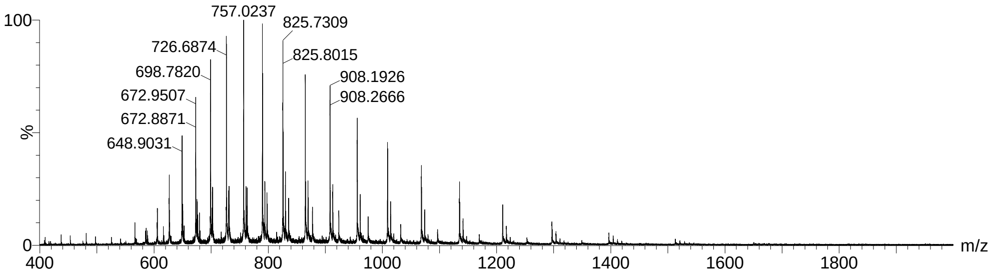

(B)

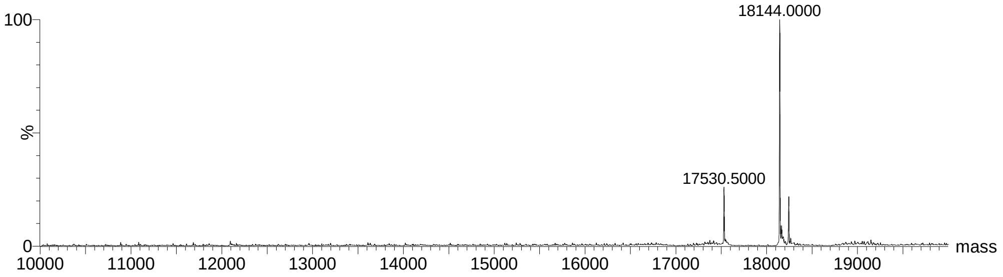

Figure S1. ESI-MS spectrum of F43Y/T67R/P88W/F138W Mb: (A) Original multiply-charged series. (B) The MaxEnt survey spectrum showing the major component. Calculated molecular weight of apo-protein, 17530 Da; holo-protein, 18145 Da; Observed, 17530.5 and 18144.0 Da.

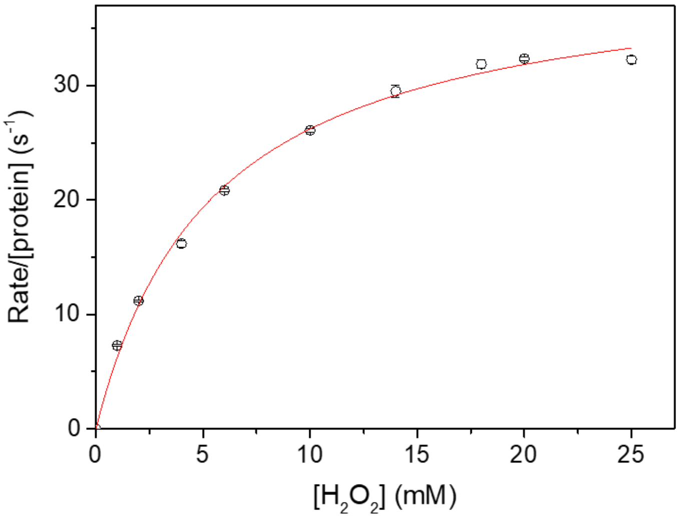

Figure S2. Initial rates of ABTS (50 μM) oxidation versus H_2O_2 concentrations catalyzed by F43Y/T67R/P88W/F138W Mb (2 μM) at pH 7.0. The data were fitted to the Michaelis–Menten equation.

Table S1. Characteristics of the textile dyes used in this study.

Dye	λ_{max} (nm)	Type	Chemical structure	Dec-l (%)
Malachite green (MG)	617	Triphenylmethane	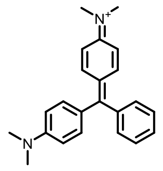	94
Brilliant blue R (BBR)	555	Triphenylmethane	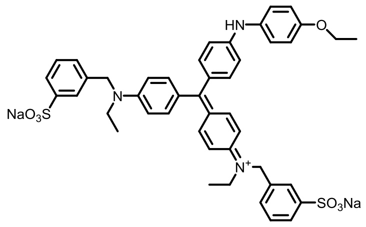	54
Reactive blue 19 (RB19)	595	Anthraquinone	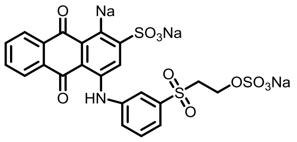	85
Reactive black 5 (RB5)	595	Double azo	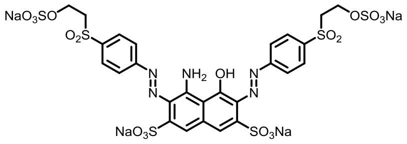	54
Amaranth (Ama)	520	Single azo	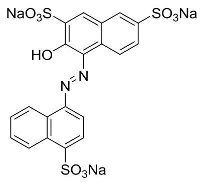	82
Reactive orange 16 (RO16)	490	Single azo	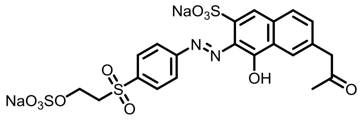	70

Dce-l (%): Decolorization level (%).
